# Pan-Cancer Analysis of the Mitophagy-Related Protein PINK1 as a Biomarker for the Immunological and Prognostic Role

**DOI:** 10.3389/fonc.2020.569887

**Published:** 2020-11-10

**Authors:** Lizhe Zhu, Wei Wu, Siyuan Jiang, Shibo Yu, Yu Yan, Ke Wang, Jianjun He, Yu Ren, Bin Wang

**Affiliations:** ^1^ Department of Breast Surgery, The First Affiliated Hospital of Xi’an Jiaotong University, Xi’an, China; ^2^ Department of Neurosurgery, The First Affiliated Hospital of Xi’an Jiaotong University, Xi’an, China

**Keywords:** PINK1, pan-cancer, prognostic, immune infiltration, biomarker

## Abstract

**Introduction:**

The PINK1 gene encodes a serine/threonine protein kinase that localizes to mitochondria and has usually been considered to protect cells from stress-induced mitochondrial dysfunction. PINK1 mutations have been observed to lead to autosomal recessive Parkinson’s disease. However, the immunological and prognostic roles of PINK1 across cancers remain unclear.

**Material and method:**

In the current study, we used multiple databases, including Oncomine, PrognoScan, Kaplan-Meier Plotter, GEPIA, TIMER, and cBioportal, to investigate the PINK1 expression distribution and its immunological and prognostic role across cancers.

**Results and discussion:**

Bioinformatics data revealed that the mRNA expression of PINK1 was downregulated in most tumors. Although there was a significant prognostic value of PINK1 expression across cancers, PINK1 played a protective or detrimental role in different kinds of cancers. Liver hepatocellular carcinoma and lung squamous cell carcinoma were selected as representative cancer types for further exploration. We found that PINK1 always played a protective role in liver hepatocellular carcinoma patients in the stratified prognostic analyses of clinicopathological characteristics. There were contradictory results between liver hepatocellular carcinoma and lung squamous cell carcinoma in the correlations of PINK1 expression with immune infiltration, including infiltration of B cells, CD8+ T cells, CD4+ T cells, macrophages, neutrophils, and dendritic cells. Furthermore, specific markers of B cells and CD8+ T cells also exhibited different PINK1-related immune infiltration patterns. In addition, there was a significant association between PINK1 copy number variations and immune infiltrates across cancers.

**Conclusion:**

The mitophagy-related protein PINK1 can work as a biomarker for prognosis and the immune response across cancers.

## Introduction

Mitochondria are double-membraned and highly dynamic organelles that play an important role in eukaryotic cells and cellular metabolism (1). Mitochondria are eliminated through a kind of autophagy known as mitophagy during certain developmental conditions or damage (2). It has been proven that mitophagy is helpful in eliminating damaged or old mitochondria to maintain cellular integrity and is beneficial for cellular homeostasis (3). In addition, mitophagy is also involved in differentiation and developmental processes, including red blood cell production and muscle differentiation (4, 5). However, the detailed role of mitophagy in tumorigenesis and tumor progression remains unknown.

The PINK1 gene encodes a serine/threonine protein kinase that localizes to mitochondria and has generally been considered to protect cells from stress-induced mitochondrial dysfunction. PINK1 mutations have been found to lead to autosomal recessive Parkinson’s disease. Research has shown that mitophagy might prevent tumorigenesis by eliminating dysfunctional mitochondria (6). Meanwhile, reports have reflected that the PINK1 kinase can regulate glycolysis and the Warburg effect by controlling mitophagy. Furthermore, PINK1 deficiency reprograms glucose metabolism through HIF1α to maintain cell proliferation and even cancer growth (7). Therefore, PINK1 deletion might be associated with carcinogenesis due to mitophagy dysfunction. However, the role of PINK1 across cancers remains unclear. There are no completed studies on the prognostic value of PINK1 across cancers.

In addition, there are also complex interactions between tumors and their microenvironment. Immune cell infiltration is an essential component of the tumor environment. The tumor microenvironment contains innate immune cells and adaptive immune cells, including neutrophils, macrophages, natural killer cells, and dendritic cells. Cancer cells are under the scrutiny of immune cells all their lives, and only if the immune cells fail to eliminate the preneoplastic cells can cancer develop and progress. Currently, different kinds of effective chemotherapies and radiotherapies partially act by activating the immune response, which restores immunosurveillance (8). With the development of immunotherapy, potential targets have been gradually discovered. For example, studies have shown that PD-L1 expression in systemic immune cell populations is a potential predictive biomarker of responses to PD-L1/PD-1 blockade therapy in lung cancer (9). Angiopoietin-2 can also work as a biomarker and target for immune checkpoint therapy (10). However, there are still patients who respond poorly to immunotherapies. Therefore, it is still necessary to further discover more specific or general immune biomarkers in cancer therapy.

In this study, we combined data from different databases, including Oncomine, PrognoScan, Kaplan-Meier Plotter, GEPIA, TIMER and cBioportal, to explore the role of PINK1 expression in prognosis and the immune response. The findings from this study indicated that PINK1 influenced the prognosis of patients with cancers and might probably *via* its interaction with infiltrating immune cells.

## Method

### Oncomine Database and the Human Protein Atlas

Oncomine is an online microarray database with large datasets and over eighty thousand samples of different kinds of cancer (11). This database was employed to analyze PINK1 mRNA expression in different kinds of human cancers. The filters included the gene name, “PINK1”, cancer vs. normal analysis, and mRNA data type. The thresholds were set as the following criteria: gene rank: 10%, fold change: 2, and p-value: 0.001. The datasets with statistically significant differences were recorded. The Human Protein Atlas is a Swedish-based program initiated in 2003 with the aim to map all the human protein in cells, tissues and organs using integration of various omics technologies, including antibody-based imaging, mass spectrometry-based proteomics, transcriptomics and systems biology. It consists of six separate parts, and each focusing on a particular aspect of the genome-wide analysis of the human proteins.

### PrognoScan, Kaplan-Meier Plotter, and GEPIA

PrognoScan is a new database for the meta-analysis of the genes with prognostic value (12). It analyzes the relationship between specific gene expression and cancer patient outcomes, such as overall survival (OS) and disease-free survival (DFS), across a large collection of publicly available cancer microarray datasets. Forest plots were drawn with GraphPad Prism 8 to summarize the survival analysis. Kaplan-Meier Plotter is an online tool for meta-analyses based on biomarker assessment (13). It is able to assess the survival of patients with different kinds of cancers based on large sample datasets. GEPIA is a newly developed interactive web server for analyzing the RNA sequencing expression data of 9,736 tumors and 8,587 normal samples from the TCGA and GTEx projects by using a standard processing pipeline (14). We used the above three tools to analyze the prognostic value of PINK1 expression in different kinds of human cancers. Hazard ratios (HRs) with 95% confidence intervals (CIs) and p-values were collected. P <0.05 was considered statistically significant.

### TIMER and R&D Systems Immune Cell Markers

The TIMER web server is a comprehensive resource for the systematic analysis of immune infiltrates across diverse cancer types (15, 16). The TIMER algorithm can estimate the abundances of six immune infiltrates, including B cells, CD4+ T cells, CD8+ T cells, neutrophils, macrophages, and dendritic cells. We used TIMER to explore the differential PINK1 expression between tumor and adjacent normal tissue across TCGA tumors. Distributions of PINK1 expression levels were adjusted to log2(TPM) data and displayed using box plots, and the statistical significance of differential expression was evaluated using the Wilcoxon test. In addition, we further analyzed the correlation between PINK1 expression and the abundance of the above six immune infiltrates across cancers. The scatterplots show the purity-corrected partial Spearman’s rho value and statistical significance. To investigate the correlation between PINK1 expression and immune cells, we explored the correlation between the specific gene expression of differential immune cells and PINK1 expression. We selected the representative gene makers of immune cells noted on the R&D Systems website (https://www.rndsystems.com/cn/resources/cell-markers/immune-cells). They were CD19, CD20, and CD3 of B cell; CD8A and CD8B of CD8+ T cell; CXCR5, ICOS and BCL-6 of follicular helper T cell; IL12RB2, WSX-1, and T-BET of T helper cell 1; CCR3, STAT6, and GATA-3 of T helper cell 2; TGFBR2, IRF4, and PU.1 of T helper cell 9; IL-21R, IL-23R, and STAT3 of T helper cell 17; CCR10 and AHR of T helper cell 22; FOXP3, CCR8, and CD25 of regulatory T cell; PD-1 and CTLA4 of T cell exhaustion; CD68 and CD11b of macrophage; NOS2 and ROS of M1 macrophage; ARG1 and MRC1 of M2 macrophage; HLA-G, CD80, and CD86 of tumor associated macrophage; CD14 and CD16 of monocyte; XCL1, KIR3DL1, and CD7 of natural killer cell; CD15 and MPO of neutrophil and CD1C and CD141 of dendritic cell. We displayed the PINK1 expression levels on the X-axis and the expression of immune cell-related gene markers on the Y-axis to draw scatterplots. All the gene expression levels in TIMER were adjusted to log2(TPM) data. Meanwhile, we explored the association of immune infiltration levels among cancers with different somatic copy number alterations (SCNAs) affecting PINK1 expression. The SCNAs in TIMER include deep deletions, arm-level deletions, diploid/normal alterations, arm-level gains and high amplifications. Box plots are presented to show the distributions of each immune subset at each copy number status in all cancers. The infiltration level for each SCNA category was compared with that for normal using a two-sided Wilcoxon rank-sum test.

### cBioPortal

The cBioPortal for Cancer Genomics contains large-scale cancer genomics data that can be visualized, downloaded and analyzed. We selected the “TCGA pancancer atlas studies” including 32 studies and 10,967 samples to further explore PINK1 alterations across cancers.

### Statistical Analysis

Our results from Oncomine database were analyzed by t-tests and showed in P values, gene rank: 10%, fold change: 2, and P value: 0.001 were used as the criteria to set thresholds. In PrognoScan, the univariate Cox regression model was used to calculate the HR and P value. In GEPIA and Kaplan-Meier Plotter, log rank test was used to calculate the HR and its P value in order to compare survival curves. Furthermore, we used Spearman correlation to analyze the correlation among different gene expression. Above all, P <0.05 was set as statistically significant if there was no special annotation.

## Result

### PINK1 mRNA and Protein Expression Level Across Cancers

**Figure 1:**
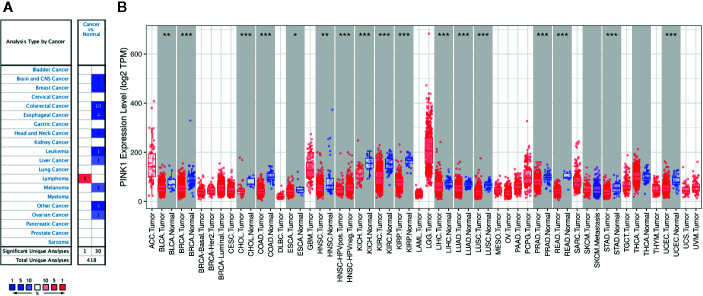
PINK1 expression levels in different types of human cancers. **(A)** The transcription levels of PINK1 in different types of human cancers. The figure is generated from ONCOMINE with exact thresholds (P-value: 0.001; fold change: 2; gene rank: top 10%). The cell number represents the dataset number that meets all of the thresholds with the color blue for under-expression and color red for over-expression. Cell color is determined by the best gene rank percentile for the analyses within the cell. **(B)** PINK1 expression levels in different types of human cancers from TCGA data in TIMER. *P < 0.05, **P < 0.01, ***P < 0.001. CNS, central nervous system; ACC, adrenocortical carcinoma; BLCA, bladder urothelial carcinoma; BRCA, breast invasive carcinoma; CESC, cervical and endocervical cancer; CHOL, cholangiocarcinoma; COAD, colon adenocarcinoma; DLBC, diffuse large B-cell lymphoma; ESCA, esophageal carcinoma; GBM, glioblastoma multiforme; HNSC, head and neck cancer; HNSC-HPVpos, head and neck cancer-HPV positive; HNSC-HPVneg, head and neck cancer-HPV negative; KICH, kidney chromophobe; KIRC, kidney renal clear cell carcinoma; KIRP, kidney renal papillary cell carcinoma; LAML, acute myeloid leukemia; LGG, lower grade glioma; LIHC, liver hepatocellular carcinoma; LUAD, lung adenocarcinoma; LUSC, lung squamous cell carcinoma; MESO, mesothelioma; OV, ovarian serous cystadenocarcinoma; PAAD, pancreatic adenocarcinoma; PCPG, pheochromocytoma and paraganglioma; PRAD, prostate adenocarcinoma; READ, rectum adenocarcinoma; SARC, sarcoma; SKCM, skin cutaneous melanoma; STAD, stomach adenocarcinoma; TGCT, testicular germ cell tumors; THCA, thyroid carcinoma; THYM, thymoma; UCEC, uterine corpus endometrial carcinoma; UCS, uterine carcinosarcoma; UVM, uveal melanoma.

**Figure 1** shows the transcription levels of PINK1 in different types of human cancers. We identified PINK1 expression across cancers and compared its mRNA expression with that in corresponding normal tissues based on data from the Oncomine database (**Figure 1A**). The results showed that PINK1 expression was lower in several cancer groups than in normal tissues, including brain, breast, colorectal, esophageal, head and neck, liver and ovarian cancers as well as leukemia and melanoma. However, the mRNA expression of PINK1 was significantly upregulated in lymphoma. The details of PINK1 expression in the above cancers are shown in **Table 1**. Over 20 unique datasets revealed that PINK1 had lower mRNA expression in different kinds of cancers than in normal tissues. In contrast, only Compagno reported that overexpression of PINK1 was found in diffuse large B cell lymphoma compared with normal tissues, with a P = 1.63E-17 and a fold change = 2.109 (**Table 1**).

**Table 1:** Datasets of PINK1 expression in pan-cancers. (ONCOMINE database).

Cancer Site	Types of Cancer vs Normal	Fold change	t-test	P-value	Dataset
Seminoma	Yolk Sac Tumor	-6.696	-24.724	1.29E-12	Korkola et al. (17)
	Embryonal Carcinoma	-3.791	-16.357	1.03E-12	
	Teratoma	-3.779	-17.980	1.53E-12	
	Mixed Germ Cell Tumor	-3.918	-16.049	4.27E-17	
	Seminoma	-3.389	-10.774	9.78E-9	
Esophagus	Esophageal Squamous Cell Carcinoma	-2.679	-12.671	9.12E-14	Hu et al. (18)
	Barrett’s Esophagus	-2.045	-4.595	2.58E-5	Hao et al. (19)
	Esophageal Adenocarcinoma	-2.019	-4.466	2.70E-4	Kimchi et al. (20)
	Esophageal Adenocarcinoma	-3.056	-11.823	6.24E-21	Kim et al. (21)
Colorectum	Rectal Adenocarcinoma	-3.871	-20.458	2.12E-29	TCGA
	Colon Adenocarcinoma	-4.081	-24.383	6.17E-28	
	Colon Mucinous Adenocarcinoma	-3.457	-13.849	4.66E-16	
	Cecum Adenocarcinoma	-4.264	-13.980	2.83E-15	
	Rectosigmoid Adenocarcinoma	-5.819	-15.085	4.25E-4	
	Colorectal Carcinoma	-2.062	-11.954	1.57E-16	Skrzypczak et al. (22)
	Colon Adenoma	-3.673	-22.192	1.89E-9	
	Colon Adenoma Epithelia	-2.394	-12.066	1.11E-8	
	Colon Adenocarcinoma	-4.095	-3.358	9.92E-4	Notterman et al. (23)
	Rectal Adenocarcinoma	-2.276	-11.718	6.51E-21	Gaedcke et al. (24)
Leukemia	Acute Myeloid Leukemia	-3.934	-7.825	2.14E-6	Stegmaier et al. (25)
Lymphoma	Diffuse Large B-Cell Lymphoma	2.109	14.511	1.63E-17	Compagno et al. (26)
Brain	Anaplastic Oligoastrocytoma	-2.669	-5.793	2.12E-4	Bredel et al. (27)
	Glioblastoma	-3.621	-10.427	4.76E-6	
	Brain Giloblastoma	-3.149	-20.046	2.49E-10	TCGA
	Glioblastoma	-2.608	-11.474	9.35E-17	Sun et al. (28)
	Glioblastoma	-2.439	-9.730	3.32E-4	Murat et al. (29)
Breast	Ductal Breast Carcinoma	-2.051	-8.074	1.63E-8	Richardson et al. (30)
Head-Neck	Head and Neck Squamous Cell Carcinoma	-2.040	-8.657	1.80E-10	Ginos et al. (31)
Liver	Hepatocellular Carcinoma	-2.060	-5.582	3.88E-6	Roessler et al. (32)
Melanoma	Cutaneous Melanoma	-2.116	-9.098	7.14E-6	Talantov et al. (33)
Ovary	Ovarian Serous Adenocarcinoma	-2.557	-7.469	1.81E-7	Yoshihara et al. (34)

Moreover, we further used TIMER to evaluate the RNA sequencing data of PINK1 in TCGA. **Figure 1B** shows the details of PINK1 expression across cancers. PINK1 expression was downregulated in many cancer types, including BLCA (bladder urothelial carcinoma), BRCA (breast invasive carcinoma), CHOL (cholangiocarcinoma), COAD (colon adenocarcinoma), ESCA (esophageal carcinoma), HNSC (head and neck cancer), KICH (kidney chromophobe), KIRC (kidney renal clear cell carcinoma), KIRP (kidney renal papillary cell carcinoma), LIHC (liver hepatocellular carcinoma), LUAD (lung adenocarcinoma), LUSC (lung squamous cell carcinoma), PRAD (prostate adenocarcinoma), READ (rectum adenocarcinoma), STAD (stomach adenocarcinoma), and UCEC (uterine corpus endometrial carcinoma). Furthermore, we assessed the PINK1 protein expression level in the human protein atlas datasets (**Supplementary Figure 1**), most cancer tissues showed moderate granular cytoplasmic positively. Collectively, PINK1 acts as a tumor suppressor in most cancer types.

### Prognostic Value of PINK1 Across Cancers

**Figure 2:**
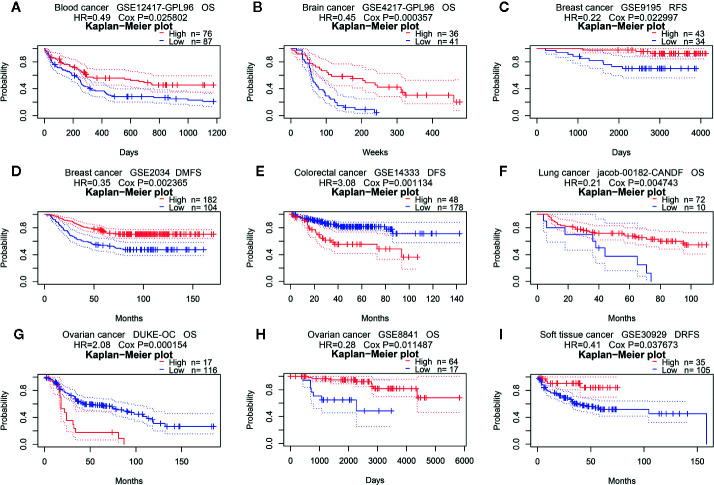
Survival analyses of PINK1 expression in pan-cancers (based on PrognoScan). **(A)** OS (n = 163) in blood cancer cohort GSE12417-GPL96. **(B)** OS (n = 77) in brain cancer cohort GSE4217-GPL96. **(C)** RFS (n = 77) in breast cancer cohort GSE9195. **(D)** DMFS (n = 286) in breast cancer cohort GSE2034. **(E)** DFS (n = 226) in colorectal cancer cohort GSE14333. **(F)** OS (n = 82) in lung cancer cohort jacob-00182-CANDF. **(G)** OS (n = 133) in ovarian cancer cohort DUKE-OC. **(H)** OS (n = 81) in ovarian cancer cohort GSE8841. **(I)** DRFS (n = 140) in soft tissue cancer cohort GSE30929. OS, overall survival; DMFS, distant metastasis free survival; RFS, relapse free survival; DFS, disease free survival; DRFS, distant recurrence free survival.

Next, we analyzed the prognostic value of PINK1 expression across cancers in PrognoScan, Kaplan-Meier Plotter, and GEPIA. First, we investigated the prognostic value of PINK1 expression in different cancer types in PrognoScan, and the detailed results are shown in **Supplementary Figure 2**. We discovered that there was a significant prognostic value of PINK1 expression in seven cancer types: colorectal, ovarian, blood, brain, breast, lung, and soft tissue cancers (**Figure 2**). The results showed that PINK1 played a protective role in five cancer types, including blood cancer (**Figure 2A**, OS: Cox P = 0.025802), brain cancer (**Figure 2B**, OS: Cox P = 0.000357), breast cancer (**Figures 2C, D**, RFS: Cox P = 0.022997; DMFS: Cox P = 0.002365), lung cancer (**Figure 2F**, OS: Cox P = 0.004743) and soft tissue cancer (**Figure 2I**, DRFS: Cox P = 0.037673). However, PINK1 played a detrimental role in colorectal cancer (**Figure 2E**, DFS: Cox P = 0.001134). Meanwhile, the role of PINK1 in ovarian cancer was controversial (**Figures 2G, H**, OS of DUKE-OC: Cox P = 0.000154; OS of GSE8841: Cox P = 0.011487).

**Figure 3:**
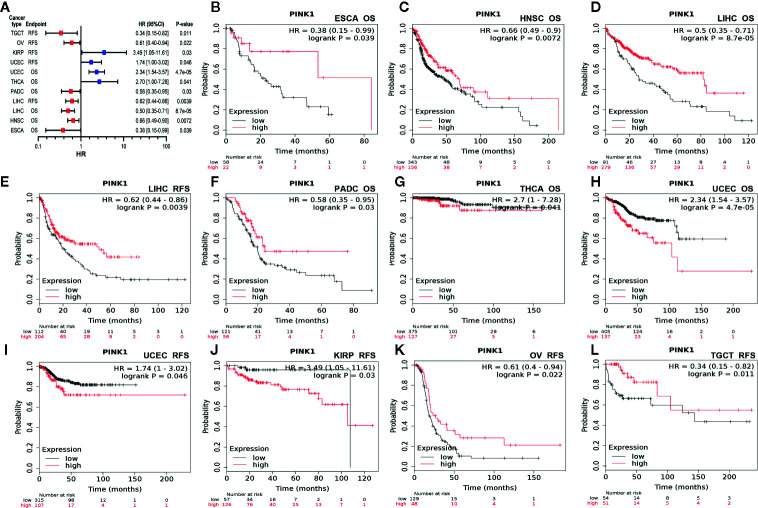
Survival analyses of PINK1 expression in different types of cancer in Kaplan-Meier plotter. **(A)** Prognostic HR of PIN1K expression in every type pf cancer. **(B)** OS of ESCA (esophageal adenocarcinoma). **(C)** OS of HNSC (head-neck squamous cell carcinoma). **(D)** OS of LIHC (liver hepatocellular carcinoma). **(E)** RFS of LIHC (liver hepatocellular carcinoma). **(F)** OS of PADC (pancreatic ductal adenocarcinoma). **(G)** OS of THCA (thyroid carcinoma). **(H)** OS of UCEC (uterine corpus endometrial carcinoma). **(I)** RFS of UCEC (uterine corpus endometrial carcinoma). **(J)** RFS of KIRP (kidney renal papillary cell carcinoma). **(K)** RFS of OV (ovarian cancer). **(L)** RFS of TGCT (testicular germ cell tumor). HR, hazard ratio; CI, confidence interval; OS, overall survival; RFS, relapse free survival.

Second, we used Kaplan-Meier Plotter to further analyze the prognostic value of PINK1 in different types of cancers (**Supplementary Table 1**). In contrast to PrognoScan, whose data mostly come from the GEO (Gene Expression Omnibus) database, Kaplan-Meier Plotter utilizes Affymetrix microarray data from TCGA. Notably, PINK1 expression was significantly correlated with nine cancer types (**Figure 3A**). Notably, we newly identified PINK1 as a protective prognostic factor in ESCA (esophageal adenocarcinoma) (OS: log rank P = 0.039) (**Figure 3B**). This discovery might challenge a previous report stating that high expression of PINK1 is a poor prognostic factor for patients with esophageal squamous cell carcinoma treated with neoadjuvant chemotherapy (35). For HNSC (head and neck squamous cell carcinoma), PINK1 was identified as a protective prognostic factor (OS: log rank P = 0.0072) (**Figure 3C**). In addition, PINK1 was also found to have a protective effect on OS and RFS in LIHC (liver hepatocellular carcinoma) (OS: log-rank P = 8.7e-05; RFS: log-rank P = 0.0039) (**Figures 3D, E**). Interestingly, we identified PINK1 as a good prognostic factor in PDAC (pancreatic ductal adenocarcinoma) (OS: log rank P = 0.03) (**Figure 3F**). This finding may further verify the previous result that PINK1 and PARK2 suppress pancreatic tumorigenesis (7). For THCA (thyroid carcinoma), PINK1 significantly affected overall survival but not relapse-free survival (OS: log-rank P = 0.041) (**Figure 3G**). Furthermore, PINK1 was newly identified as a detrimental prognostic factor for both OS and RFS in UCEC (uterine corpus endometrial carcinoma) (OS: log rank P = 4.7e-05; RFS: log rank P = 0.046) (**Figures 3H, I**). In addition, for kidney cancer, PINK1 had a detrimental effect on RFS in only KIRP (kidney renal papillary cell carcinoma) (RFS: log rank P = 0.03) (**Figure 3J**). For both OV (ovarian cancer) and TGCT (testicular germ cell tumor), PINK1 significantly influenced RFS but not OS (RFS: log-rank P = 0.022; RFS: log-rank P = 0.011) (**Figures 3K, L**).

Third, in addition to our analyses of the microarray data of PINK1 in PrognoScan and Kaplan-Meier Plotter, we used the online tool GEPIA to analyze RNA sequencing data from TCGA. We analyzed the role of PINK1 in 33 kinds of cancer (**Supplementary Table 2**). Interestingly, PINK1 had a significant overall effect on cancers (OS: total number = 9,500, HR = 0.69, log-rank P = 0; DFS: total number = 9,500, HR = 0.82, log-rank P = 1.4e-07). Compared with low expression of PINK1, high expression of PINK1 was positively correlated with better OS in ACC (adrenocortical carcinoma) and MESO (mesothelioma), DFS in DLBC (lymphoid neoplasm diffuse large B-cell lymphoma), and both OS and DFS in KIRC (kidney renal clear cell carcinoma) and KIRP (kidney renal papillary cell carcinoma). Meanwhile, LIHC (liver hepatocellular carcinoma) outcome was also found to have a positive correlation with PINK1 expression (DFS: HR = 0.75, P-value = 0.057). In contrast, a high expression level of PINK1 was negatively correlated with both OS and DFS in HNSC (head and neck squamous cell carcinoma) and LUSC (lung squamous cell carcinoma). In other cancer types, PINK1 expression had no significant effect on prognosis.

### Stratified Prognostic Analyses of PINK1 Expression in LIHC and LUSC

**Figure 4:**
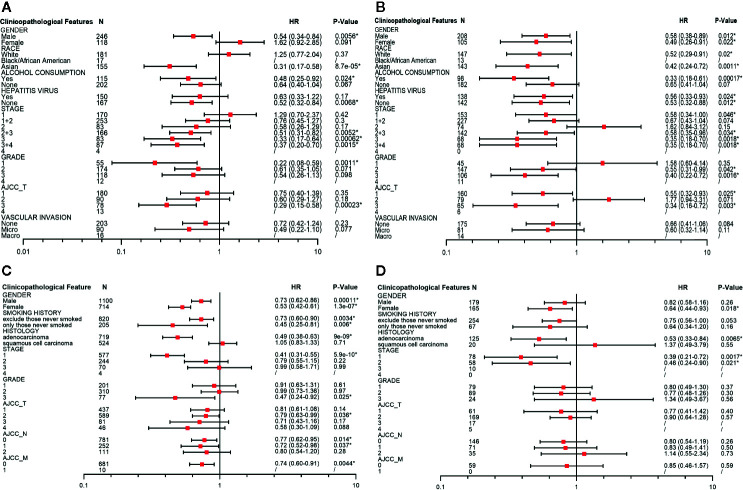
Survival analyses of PINK1 with different clinicopathologic features in liver hepatocellular carcinoma and lung cancer. **(A)** Correlation of PINK1 mRNA expression with OS in liver hepatocellular carcinoma. **(B)** Correlation of PINK1 mRNA with RFS in liver hepatocellular carcinoma. **(C)** Correlation of PINK1 mRNA expression with OS in lung cancer. **(D)** Correlation of PINK1 mRNA with PPS in lung cancer. *P < 0.05. HR, hazard ratio; OS, overall survival; RFS, relapse free survival; PPS, post progression survival.

To identify the potential mechanisms of PINK1 expression across cancers, we used LIHC as an example. We used Kaplan-Meier Plotter to explore the correlation between PINK1 expression and several clinicopathological characteristics in LIHC. The results showed that PINK1 played a protective role in LIHC patients with the following clinicopathological characteristics: male sex (OS: P = 0.0056; RFS: P = 0.012), Asian race (OS: P = 8.7e-05; RFS: P = 0.0011), alcohol consumption (OS: P = 0.024; RFS: P = 0.00017), no hepatitis virus infection (OS: P = 0.0068; RFS: P = 0.012), pathology stage 3 (OS: P = 0.00062; RFS: P = 0.0018) and AJCC stage 3 (OS: P = 0.00023; RFS: P = 0.003). Furthermore, for RFS, PINK1 expression also had a significantly positive effect on female (RFS: P = 0.022), white (RFS: P = 0.02), hepatitis virus-infected (RFS: P = 0.024), pathology stage 1 (RFS: P = 0.046) and AJCC stage 1 (RFS: P = 0.025) LIHC patients (**Figure 4**).

We also conducted the detailed evaluation of PINK1 expression and clinicopathological characteristics in lung cancer. **Figures 4C, D** showed that PINK1 played a protective role in lung cancer patients with the following clinicopathological characteristics: female sex (OS: P = 1.3e-07; PPS: P = 0.018), adenocarcinoma (OS: P = 9e-09; PPS: P = 0.0065) and pathology stage 1(OS: P = 5.9e-10; PPS: P = 0.0017). In addition, for OS, PINK1 expression also had a significantly positive effect on male (OS: P = 0.00011), excluded those never smoked and only those never smoked of smoking history (OS: P = 0.0034; OS: P = 0.006), grade 3 (OS: P = 0.025), AJCC T stage 2 (OS: P = 0.036), AJCC N stage 0 and 1 (OS: P = 0.014; P = 0.037) and AJCC M stage 0 (OS: P = 0.0044). The detailed prognostic values of different clinicopathologic characteristics in LIHC and LUSC are listed in **Figure 4**.

### Conflicting Correlations Between PINK1 Expression and Immune Infiltration in LIHC and LUSC

Determining the interactions between the host immune system and tumor is essential to discover prognostic biomarkers, as well as for reducing drug resistance and developing new cancer therapies (16). It is well known that immune infiltration in the tumor microenvironment can affect patient survival. We also verified that PINK1 might play a prognostic role across cancers. Furthermore, we analyzed the correlation between PINK1 expression and immune infiltration. We used the online TIMER database to explore the correlation between PINK1 expression and the infiltration levels of six immune cell types across cancers. The results showed that PINK1 was significantly correlated with tumor purity in 18 out of 39 cancer types. Meanwhile, the correlations between PINK1 expression and the infiltration levels of B cells, CD8+ T cells, CD4+ T cells, macrophages, neutrophils and dendritic cells were significant in 13, 15, 20, 23, 15, and 21 cancer types, respectively (**Supplementary Figure 3**).

**Figure 5:**
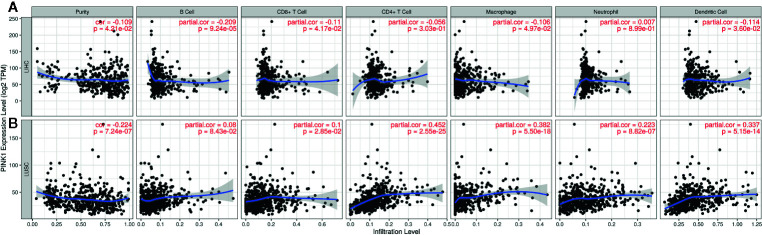
The correlation between PINK1 expression and immune infiltration level in LIHC and LUSC. **(A)** Correlation of PINK1 expression with tumor purity and infiltrating levels of B cell, CD8+ T cell, CD4+ T cell, macrophage, neutrophil and dendritic cell in LIHC. **(B)** Correlation of PINK1 expression with tumor purity and infiltrating levels of B cell, CD8+ T cell, CD4+ T cell, macrophage, neutrophil, and dendritic cell in LUSC. P < 0.05 is considered as statistically significant. LIHC, liver hepatocellular carcinoma; LUSC, lung squamous cell carcinoma.

The GEPIA and TIMER databases are founded on TCGA data. Therefore, based on the findings from GEPIA, LIHC, and LUSC were selected to analyze PINK1 expression and immune infiltration. LIHC represents a cancer in which patients had good survival with high PINK1 expression, while LUSC represents a cancer in which patients had poor survival with high PINK1 expression. **Figure 5** shows that PINK1 had significant correlations with tumor purity in both LIHC and LUSC (LIHC: R = -0.109, P = 4.21e-02; LUSC: R = -0.224, P = 7.24e-07). For LIHC, PINK1 expression was significantly negatively correlated with the infiltration levels of four kinds of immune cells: B cells (R = -0.209, P = 9.24e-05), CD8+ T cells (R = -0.11, P = 4.17e-02), macrophages (R = -0.106, P = 4.97e-02) and dendritic cells (R = -0.114, P = 3.60e-02) (**Figure 5A**). In contrast, the PINK1 expression level was significantly positively correlated with the infiltration level of CD8+ T cells (R = 0.1, P = 2.85e-02), CD4+ T cells (R = 0.452, P = 2.55e-25), macrophages (R = 0.382, P = 5.50e-18), neutrophils (R = 0.223, P = 8.82e-07), and dendritic cells (R = 0.337, P = 5.15e-14) in LUSC (**Figure 5B**). Taken together, the results above reflect that PINK1 expression might affect cancer patient survival by influencing immune infiltration in the tumor microenvironment.

### Correlation Between PINK1 Expression and Representative Immune Markers in LIHC and LUSC

The above analyses revealed that PINK1 expression might play an important role in immune infiltration in the tumor microenvironment. We then performed a deeper investigation of the relationship between PINK1 expression and representative immune markers of several immune cells. The immune cells included B cells, CD8+ T cells, follicular helper T cells, T helper 1 cells, T helper 2 cells, T helper 9 cells, T helper 17 cells, T helper 22 cells, regulatory T cells, exhausted T cells, macrophages, M1 macrophages, M2 macrophages, tumor-associated macrophages, monocytes, natural killer cells, neutrophils, and dendritic cells. We selected LIHC and LUSC to explore the relationships in TIMER. Adjustment for tumor purity was performed for accuracy of correlation. PINK1 expression was significantly correlated with 34 of the 45 cell types in LIHC and with 31 of the 45 cell types in LUSC (**Table 2**). **Table 2** shows that the correlation of PINK1 expression with these immune cell types was different in LIHC and LUSC. In LIHC, PINK1 expression was significantly correlated with B cell- and CD8+ T cell-specific markers, while LUSC showed no correlations of PINK1 with these cells. In addition, PINK1 expression was more strongly correlated with tumor-associated macrophage markers, such as HLA-G, CD80, and CD86, in LUSC than in LIHC. These analyses further confirmed that PINK1 expression plays an important role in immune infiltration in cancers such as LIHC and LUSC. These results might contribute to the understanding of the survival differences seen in cancer patients.

**Table 2:** The correlation between PINK1 expression and immune cells related gene markers. (TIMER database).

Cell type	Gene marker	LIHC				LUSC			
		None		Purity		None		Purity	
		Cor	P	Cor	P	Cor	P	Cor	P
B cell	CD19	-0.124	*	-0.191	***	0.146	**	0.029	0.530
	CD20	-0.138	**	-0.255	***	0.100	*	-0.019	0.673
	CD38	-0.047	0.368	-0.111	*	0.088	*	0.008	0.856
CD8+ T Cell	CD8A	-0.045	0.387	-0.124	*	0.127	**	0.052	0.256
	CD8B	-0.070	0.179	-0.145	**	0.061	0.176	0.009	0.837
Tfh	CXCR5	-0.143	**	-0.237	***	0.239	***	0.147	**
	ICOS	-0.171	**	-0.255	***	0.187	***	0.089	0.051
	BCL-6	-0.001	0.989	-0.007	0.898	0.006	0.885	0.036	0.434
Th1	IL12RB2	0.076	0.144	0.044	0.418	0.137	**	0.098	*
	WSX-1	-0.109	*	-0.167	**	0.259	***	0.227	***
	T-BET	0.028	0.586	-0.043	0.425	0.232	***	0.155	**
Th2	CCR3	-0.044	0.403	-0.084	0.119	0.226	***	0.168	***
	STAT6	0.156	**	0.160	**	0.138	**	0.140	**
	GATA-3	-0.053	0.312	-0.133	*	0.366	***	0.330	***
Th9	TGFBR2	0.184	***	0.164	**	0.499	***	0.457	***
	IRF4	-0.093	0.073	-0.186	**	0.220	***	0.126	**
	PU.1	-0.040	0.442	-0.144	**	0.366	***	0.292	***
Th17	IL-21R	-0.117	*	-0.214	***	0.291	***	0.212	***
	IL-23R	-0.056	0.286	-0.073	0.178	0.130	**	0.060	0.190
	STAT3	0.163	**	0.139	*	0.222	***	0.206	***
Th22	CCR10	-0.125	*	-0.136	*	0.325	***	0.284	***
	AHR	0.130	*	0.106	*	0.099	*	0.082	0.074
Treg	FOXP3	0.094	0.072	0.065	0.229	0.308	***	0.234	***
	CCR8	-0.097	0.062	-0.161	**	0.254	***	0.179	***
	CD25	-0.048	0.353	-0.130	**	0.192	***	0.103	*
T cell exhaustion	PD-1	-0.192	***	-0.273	***	0.224	***	0.149	**
	CTLA4	-0.235	***	-0.317	***	0.159	***	0.060	0.191
Macrophage	CD68	-0.025	0.628	-0.089	0.098	0.300	***	0.228	***
	CD11b	0.059	0.255	0.004	0.934	0.369	***	0.306	***
M1	NOS2	0.194	***	0.191	***	-0.038	0.401	-0.040	0.382
	ROS	0.204	***	0.192	***	0.281	***	0.218	***
M2	ARG1	0.202	***	0.181	**	-0.040	0.375	-0.049	0.289
	MRC1	0.296	***	0.254	***	0.357	***	0.293	***
TAM	HLA-G	0.148	**	0.124	*	0.237	***	0.187	***
	CD80	-0.043	0.405	-0.114	*	0.212	***	0.136	**
	CD86	-0.024	0.647	-0.115	*	0.286	***	0.202	***
Monocyte	CD14	0.305	***	0.280	***	0.358	***	0.285	***
	CD16	0.077	0.141	0.016	0.772	0.262	***	0.189	***
NK	XCL1	-0.113	*	-0.137	*	-0.076	0.088	-0.044	0.337
	KIR3DL1	0.162	**	0.167	**	0.180	***	0.145	**
	CD7	-0.169	**	-0.237	***	0.218	***	0.132	**
Neutrophil	CD15	-0.155	**	-0.180	**	0.213	***	0.202	***
	MPO	0.127	*	0.102	0.059	0.242	***	0.185	***
DC	CD1C	-0.054	0.298	-0.115	*	0.235	***	0.128	**
	CD141	0.145	**	0.101	0.060	0.096	*	0.087	0.058

*P < 0.05, **P <0.01, ***P < 0.001.

LIHC, liver hepatocellular carcinoma; LUSC, lung squamous cell carcinoma; Tfh, follicular helper T cell; Th, T helper cell; Treg, regulatory T cell; TAM, tumor associated macrophage; NK, natural killer cell; DC, dendritic cell; None, correlation without adjustment; Purity, correlation adjusted for tumor purity; Cor, R value of Spearman’s correlation.

### Association Between PINK1 Copy Number Variations and Immune Infiltrates Across Cancers

**Figure 6:**
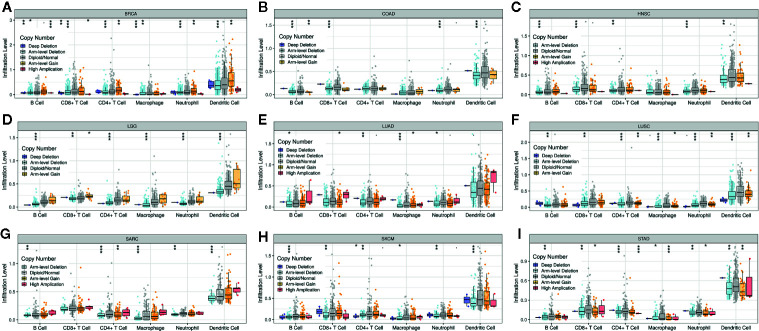
The association between PINK1 copy number variations and immune infiltrates in **(A)** BRCA (breast invasive carcinoma), **(B)** COAD (colon adenocarcinoma), **(C)** HNSC (head and neck cancer), **(D)** LGG (lower grade glioma), **(E)** LUAD (lung adenocarcinoma), **(F)** LUSC (lung squamous cell carcinoma), **(G)** SARC (sarcoma), **(H)** SKCM (skin cutaneous melanoma), **(I)** STAD (stomach adenocarcinoma). *P < 0.05, **P < 0.01, ***P < 0.001.

**Supplementary Figure 4** shows that the PINK1 gene is frequently altered in across cancers. The association between PINK1 copy number variations and immune infiltrates in different kinds of cancer was investigated. **Figure 6** shows nine of the most significant relationships between the changes in PINK1 copy number variations and six types of immune infiltrates in all cancers. In particular, deletion of PINK1 was associated with substantially lower levels of six immune infiltrates in breast invasive carcinoma, colon adenocarcinoma, head and neck cancer, lower grade glioma, lung adenocarcinoma, lung squamous cell carcinoma, sarcoma, skin cutaneous melanoma, and stomach adenocarcinoma. However, deletion of PINK1 was associated with substantially higher CD4+ T cell, neutrophil and dendritic cell counts in breast invasive carcinoma. These findings might suggest the potential mechanism by which PINK1 alterations predict the response to immune therapy.

## Discussion

As an evolutionarily conserved cellular process, mitophagy selectively eliminates dysfunctional mitochondria by directing them to the autophagosome and degrading them. However, when mitophagy is dysregulated, mitochondria are damaged and accumulate, causing carcinogenesis and cancer progression (36). PINK1 does not affect the mitophagy process directly (37). Mitochondrial depolarization recedes PINK1 cleavage and causes PINK1 accumulation at the outer membrane of mitochondria by the TOM/TIM complex. The ubiquitin-tagged outer mitochondrial membrane proteins bind to diverse LC3 binding regions that involve different kinds of autophagy adaptors, such as OPTN, NBR1, and TAX1BP1 (38, 39). In addition to the role of ubiquitin-dependent receptors in mitophagy, PINK1 also plays an extensive role in cancer. Research has shown that PINK1 can work as a regulator of the Warburg effect and a negative regulator of glioblastoma growth, and the loss of PINK1 contributes to the Warburg effect through ROS-dependent stabilization of HIF1A (40). Silencing of PINK1 inhibited the proliferation of lung cancer cells and blocked the cell cycle (41). However, the role of PINK1 in metastasis in different cancers has not yet been widely studied. Whether PINK1 expression influences cancer patient survival remains unknown. Our analyses were all based on human datasets. According to our study, we found that PINK1 expression was lower in several cancers, including brain, breast, colorectal, esophageal, head and neck, liver, and ovarian cancers as well as leukemia and melanoma, at the mRNA level. Both the Oncomine and TIMER databases showed low expression levels of PINK1 in most cancer types. This might indicate that PINK1 plays a protective role across cancers. However, this was a preliminary assumption. To confirm the role of PINK1 in cancers, we analyzed the prognosis of patients according to PINK1 expression in different kinds of cancer with data from various databases.

Our analyses showed that PINK1 played a protective role in five cancer types, including blood cancer, brain cancer, breast cancer, lung cancer, and soft tissue cancer. However, PINK1 played a detrimental role in colorectal cancer, and the role of PINK1 in ovarian cancer was controversial. Unlike PrognoScan, which uses data mostly from GEO, the Kaplan-Meier Plotter utilizes Affymetrix microarray data from TCGA. PINK1 expression was significantly correlated with nine cancer types and played a protective role in ESCA, HNSC, LIHC, PADC, OV, and TGCT. The results were not completely consistent. These discrepancies in PINK1 expression in different kinds of cancer might be due to the heterogeneity of data collection as well as a result of the presence of specific biological properties in each cancer type. However, the PINK1 expression in LIHC and LUSC showed consistency in the three databases. We further explored the correlation between PINK1 expression and several clinicopathological characteristics in LIHC and lung cancer. Interestingly, the results showed that PINK1 always played a protective role in LIHC patients with the following clinicopathological characteristics: male sex, Asian race, alcohol consumption, no hepatitis virus infection, pathology stage 3, and AJCC stage 3. The similar results could also be got from lung cancer. Taken together, our results revealed that the role of PINK1 might be context dependent and could vary among different cancers. PINK1 played a protective role in most cancers. However, the discrepant PINK1 expression in different database might reflect the different underlying mechanisms which need to be further verified by experiments. We all know that cancer is a highly heterogeneous disease, and the crosstalk within the tumor is also very diverse. For example, a drug that is effective for a certain gene in a certain cancer is also effective for other cancers that express that gene. In response to this situation, researchers believe that the molecular signals that stem from the same genes in different cancer cells are different, leading to different therapeutic effects. Generally, the gene changes in the same cancer may be different. Similarly, the expression and function of the same gene in different cancers are also different (42, 43). Above all, these findings could provide a view on the utility of the mitophagy-related protein PINK1 as a prognostic factor across cancers.

Scientific observations have exemplified the critical influence of mitochondrial metabolism on malignant transformation and tumor progression (44–46). Defects in immunity also contribute to carcinogenesis, cancer progression, and poor cancer treatment efficacy. Take anti-PD1 immunotherapy as an example, in most advanced cancers, except Hodgkin lymphoma (which has high PD-L1/L2 expression) and melanoma (which has high tumor mutational burden), the objective response rate with anti-PD-1/PD-L1 monotherapy is only ~20%. It suggested that the roles of PD-1/PD-L1 in immune suppression remain to be better defined and important immune regulatory mechanisms within or outside of the PD-1/PD-L1 network need to be discovered (47). This concept prompted us to elucidate the immunological role of the mitophagy-related protein PINK1 across cancers to determine whether the immune system plays any role in the prognostic value of PINK1.

There have been several studies about the role of PINK1 expression in tumor immune function. Sun et al. discovered that a lack of PINK1 alters glial innate immune responses and enhances nitric oxide-mediated neuron death (48). Basit F verified that dendritic cells required PINK1-mediated phosphorylation of BCKDE1α to promote fatty acid oxidation for immune function (48). None of the studies focused on the relationship between PINK1 expression and immune infiltration in cancer patients. We used the online TIMER database to explore the correlation between PINK1 expression and the levels of six kinds of immune infiltrates across cancers. In our study, results showed that PINK1 had significant correlations with tumor purity in both LIHC and LUSC (LIHC: P = 4.21e-02; LUSC: P = 7.24e-07), which indicating their comparative enrichment in tumor cells and may be attributable to the enrichment patterns of PINK1 in the tumor microenvironment. The results showed that PINK1 was significantly correlated with six different immune infiltrates, B cells, CD8+ T cells, CD4+ T cells, macrophages, neutrophils and dendritic cells, across cancers. In addition to the contradictory results of the prognostic value of PINK1 expression in LIHC and LUSC, there were also conflicting results in the correlations between PINK1 expression and immune infiltrates in LIHC and LUSC. For LIHC, PINK1 expression was significantly negatively correlated with immune infiltration levels in four kinds of immune cells: B cells, CD8+ T cells, macrophages and dendritic cells, while PINK1 was positively correlated with these cell types in LUSC. We further explored the correlation between PINK1 expression and representative immune markers in LIHC and LUSC, and PINK1 expression was more strongly correlated with tumor-associated macrophage markers such as HLA-G, CD80, and CD86 in LUSC than in LIHC. Moreover, PINK1 expression was significantly correlated with B cell- and CD8+ T cell-specific markers in LIHC, while no correlation of PINK1 expression with these markers was shown in LUSC. Meanwhile, the increase of CD4+ T cells and neutrophils would result in the poorer prognosis of LUSC than LIHC. Several studies, though not comprehensively elucidated, showed consistency with our analysis. Various CD4+ T cells, including regulatory T (Treg) and T helper 17 (Th17) CD4+ T cell have been observed to mechanistically promote tumorigenesis, cancer progression and metastasis through immunosuppressive and pro-inflammatory functions. Specifically, Tregs contributes to poorer prognosis of LUSC by inducing immunosuppression through contact-dependent mechanisms in LUSC such as the expression of cytotoxic T-lymphocyte-associated protein 4 (CTLA-4), programmed cell death 1 (PD-1), programmed death-ligand 1 (PD-L1) et al. Tregs also influences the tumor microenvironment during the progression of LUSC by inhibiting CD8+ T cell-mediated anti-tumor immunity and resulting in tumor cell death. Th17 contributes to poorer prognosis of LUSC by expressing the transcription factors RORγt/RORC2 (mouse/human) and RORα, which drive Th17 differentiation and produce pro-inflammatory cytokines IL-17, which has been shown to promote tumor growth by increasing angiogenesis, metastasis and macrophage infiltration into tumors. Th17 cells also produces other cytokines in addition to IL-17, including IL-22, which contributes to angiogenesis and metastasis (49). For neutrophils, Marta et al. had reported that in LUSC, anti-angiogenic therapy increased the expression of stem cell maker CD15 which also worked as a neutrophil related gene, which would cause disease progression and also induce tumor proliferation (50). Another neutrophil related gene MPO have been found to be released from neutrophils in lung tissue in response to exposure to various pulmonary insults. MPO had been also shown to activate an intermediate metabolite of B(a)P to the highly reactive and carcinogenic B(a)P diol epoxide and to enhance the binding of B(a)P diol to lung DNA *in vitro* and finally lead to disease progression (51). These results confirmed our hypothesis that PINK1 expression in LIHC and LUSC correlates with immune cell in different manners, which might help explain the differences in patient survival. With the development of scientific research, the tumor environment including immune cell infiltration could help us elucidate the mechanisms behind tumor development. However, we could only figure out the significant correlations between immune cell infiltration and PINK1 expression in tumors, the cause-effect relationship was hard to established. Recent studies have already showed some possible mechanisms deciphering why PINK1 expression correlated with different prognoses and immune infiltration in pan-cancer. It was acknowledged that the immune system prevents cancer from developing by activating T cells and macrophages to attack tumor cells. However, once the tumor progresses beyond this early stage, the immune TME would transform to support cancer cells, promote tumor progression and suppress immune cell mediated cytotoxicity (52, 53). Our results also find that the immune infiltration levels of antigen presenting cells such as macrophages and B cells are significantly correlated with the PINK1 expression level in cancers. And there were also researchers reported the mitophagy could reduce CD8+ T cell activation. Besides, the mitophagy was also involved in the development and differentiation of immune cells such like NK cells, macrophages and T cells (52). In addition, we also find out that there was a strong association between PINK1 copy number variation and immune infiltrates across cancers. All of the above findings revealed that the correlation between PINK1 expression and immune infiltration exists but various in different types of cancer, it might contribute to patient survival by influencing immune infiltration in the tumor microenvironment.

Although we performed a substantial exploration of the role of PINK1 expression in both prognostic and immunological aspects, there were some limitations of our analysis. First, multiple analyses based on diverse databases could provide a large amount of data as well as data heterogeneity. Therefore, some of the results were conflicting. Second, our analyses were based on pancancer data in patients, and it might be difficult to perform experiments to prove all the ideas at the same time. Further *in vivo*/*in vitro* experiments and even clinical trials are needed to verify the highlights from our bioinformatics analysis. Third, although we found that PINK1 expression correlated well with immune infiltration and patient survival, we did not find that PINK1 affects cancer patient prognosis *via* immune infiltration. This hypothesis remains to be verified.

## Conclusion

In conclusion, the mitophagy-related protein PINK1 is significantly correlated with prognosis and immune infiltration across cancers, especially in LIHC and LUSC. PINK1 might work as a new biomarker for prognostic prediction and immune cell infiltration across cancers in the future.

## Data Availability Statement

The datasets presented in this study can be found in online repositories. The names of the repository/repositories and accession number(s) can be found in the article/**Supplementary Material**.

## Author Contributions

LZ: Collection and/or assembly of data, data analysis and interpretation, manuscript writing, methodology, and software. WW: Collection and/or assembly of data, data analysis and interpretation, manuscript writing and editing. SJ: Data analysis, manuscript writing and interpretation. SY: Manuscript writing and project administration. YY: Manuscript writing and project administration. KW: Manuscript writing. JH: Conception/design. YR: Conception/design, supervision and editing. BW: Conception/design, supervision, and editing. All authors contributed to the article and approved the submitted version.

## Funding

This study was supported by grants from National Natural Science Fund of China (number 81702633).

## Conflict of Interest

The authors declare that the research was conducted in the absence of any commercial or financial relationships that could be construed as a potential conflict of interest.
