# Supplementary material for: Pan-Cancer Analysis of the Mitophagy-Related Protein PINK1 as a Biomarker for the Immunological and Prognostic Role
Source: Front Oncol. 2020 Nov 10;10:569887. doi: 10.3389/fonc.2020.569887 (PMC7683787; doi:10.3389/fonc.2020.569887)
Supplement: Supplementary file 1 [file DataSheet_1.pdf]

Supplementary Material

1 Supplementary Figures and Tables

1.1 Supplementary Figures

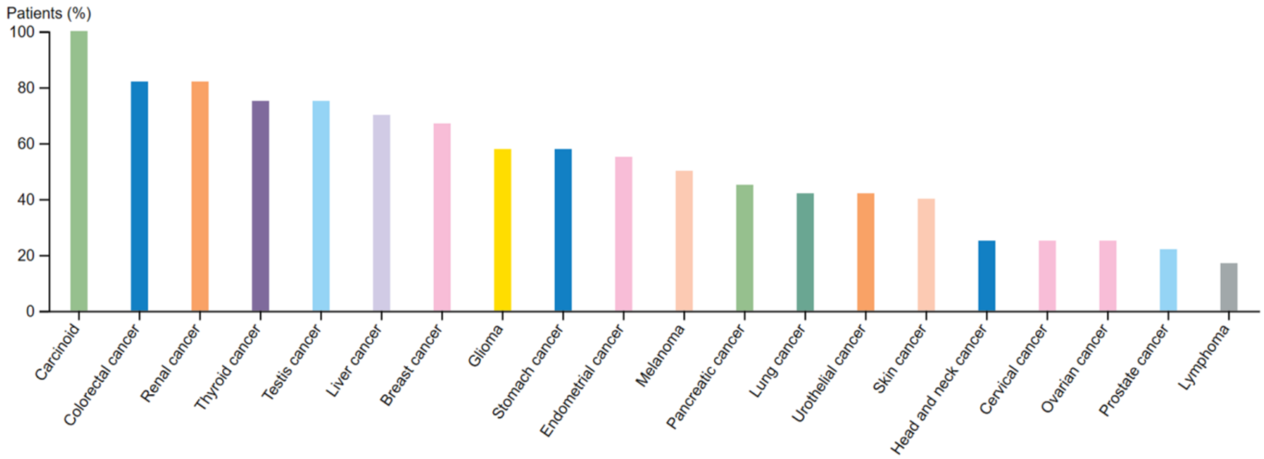

Supplementary Figure 1. Pan-cancer analysis of PINK1 protein expression level across cancers in the Human Protein Atlas.

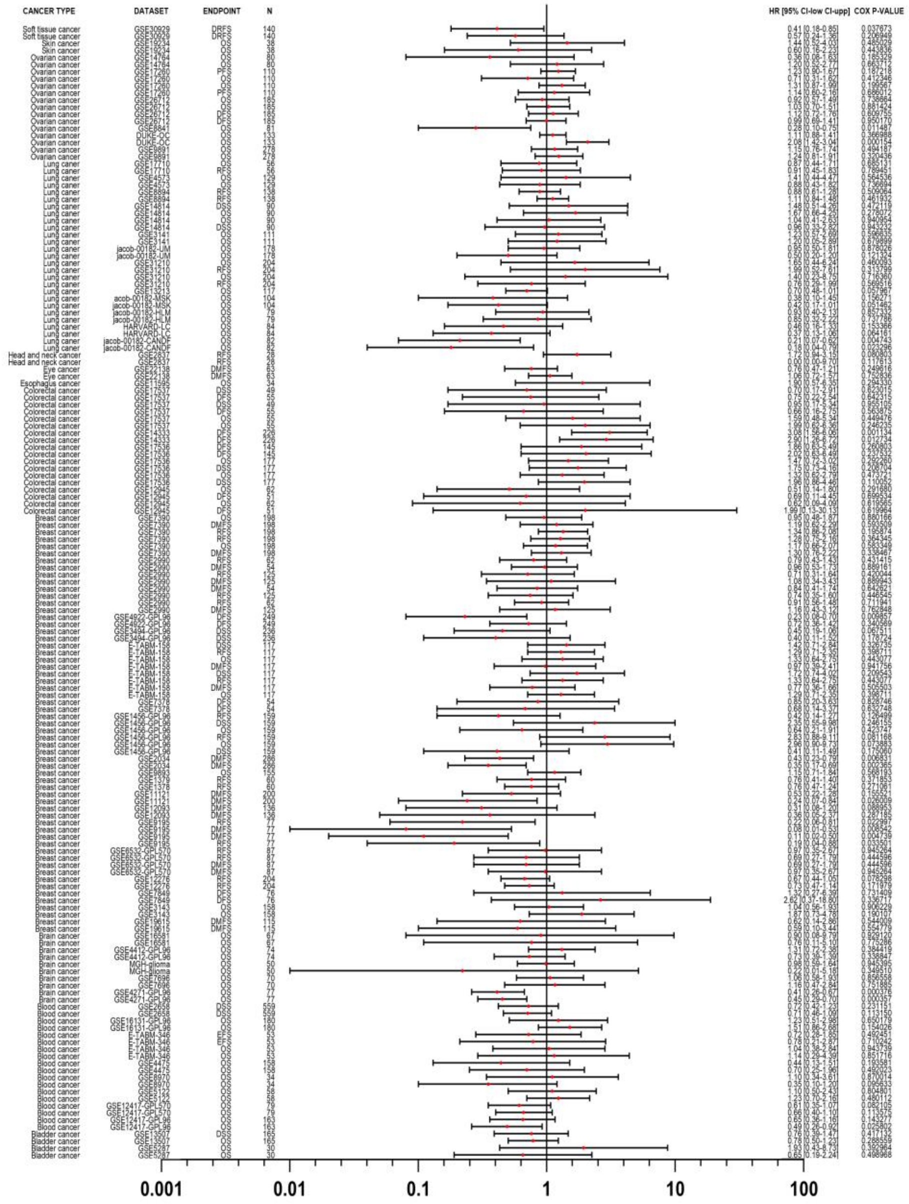

Supplementary Figure 2. Pan-cancer prognostic analysis of PINK1 expression in different datasets of cancers in PrognoScan. The red circle represents hazard ratio. Abbreviations: OS, overall survival;

DSS, disease specific survival; EFS, event free survival; DMFS, distant metastasis free survival; RFS, relapse free survival; DFS, disease free survival; DRFS, distant recurrence free survival.

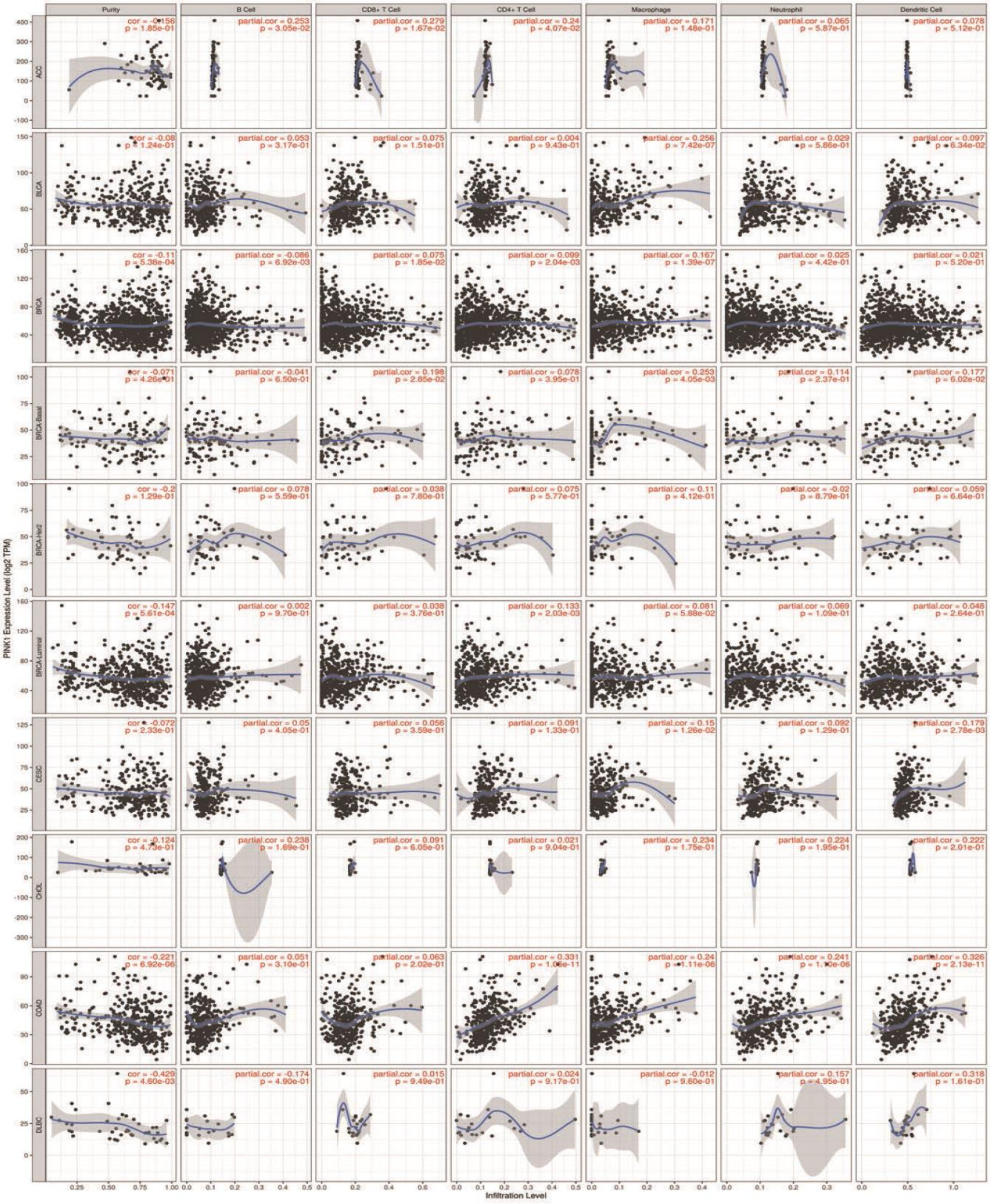

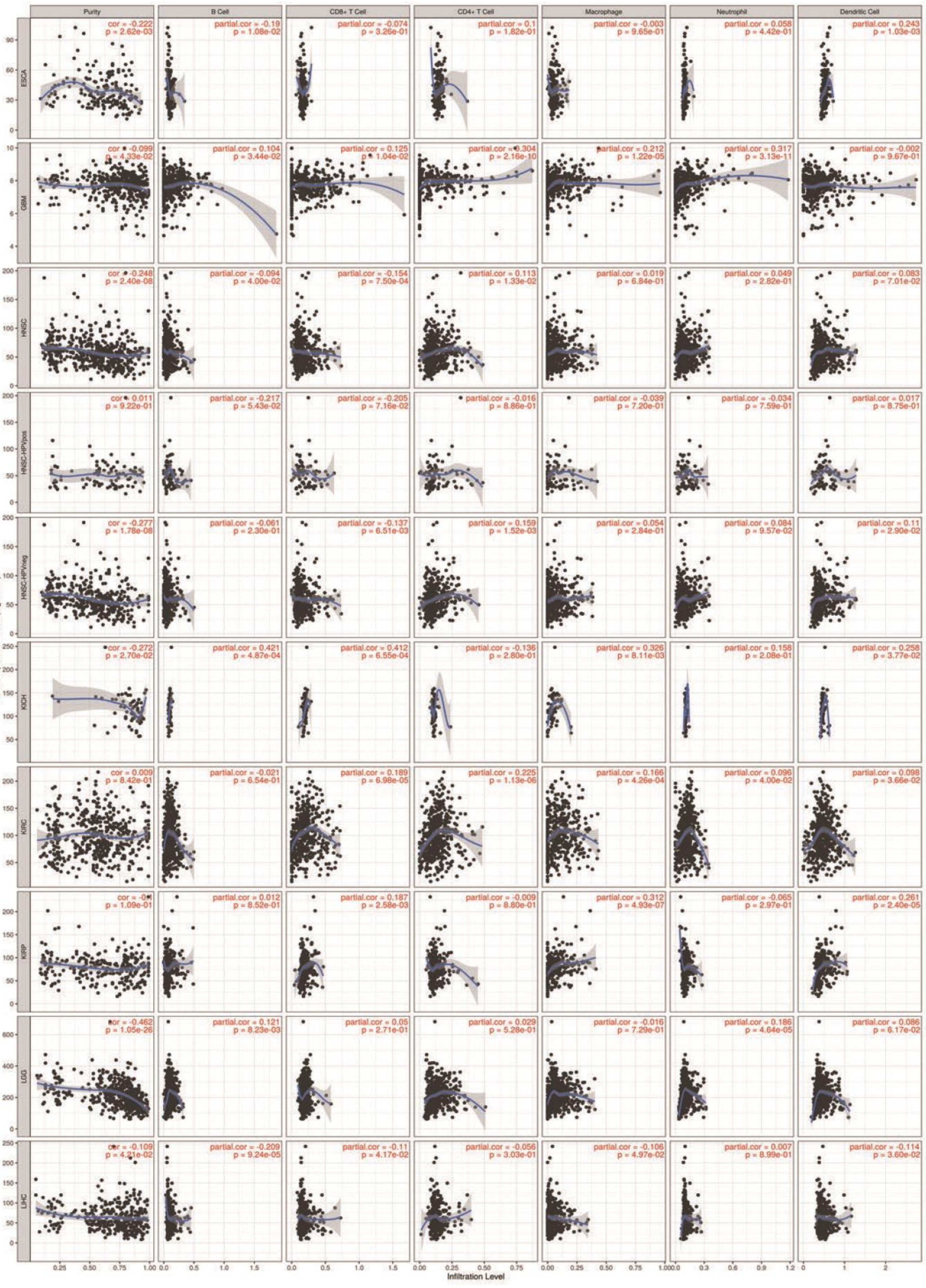
Purity
B Cell
CD8+ T Cell
CD4+ T Cell
Macrophage
Neutrophil
Dendritic Cell
ESCA
GBM
HNSC
HNSC-HPVpos
HNSC-HPVneg
KICH
KIRC
KIRP
LGG
LIHC
PINK1 Expression Level (log2 TPM)
Infiltration Level

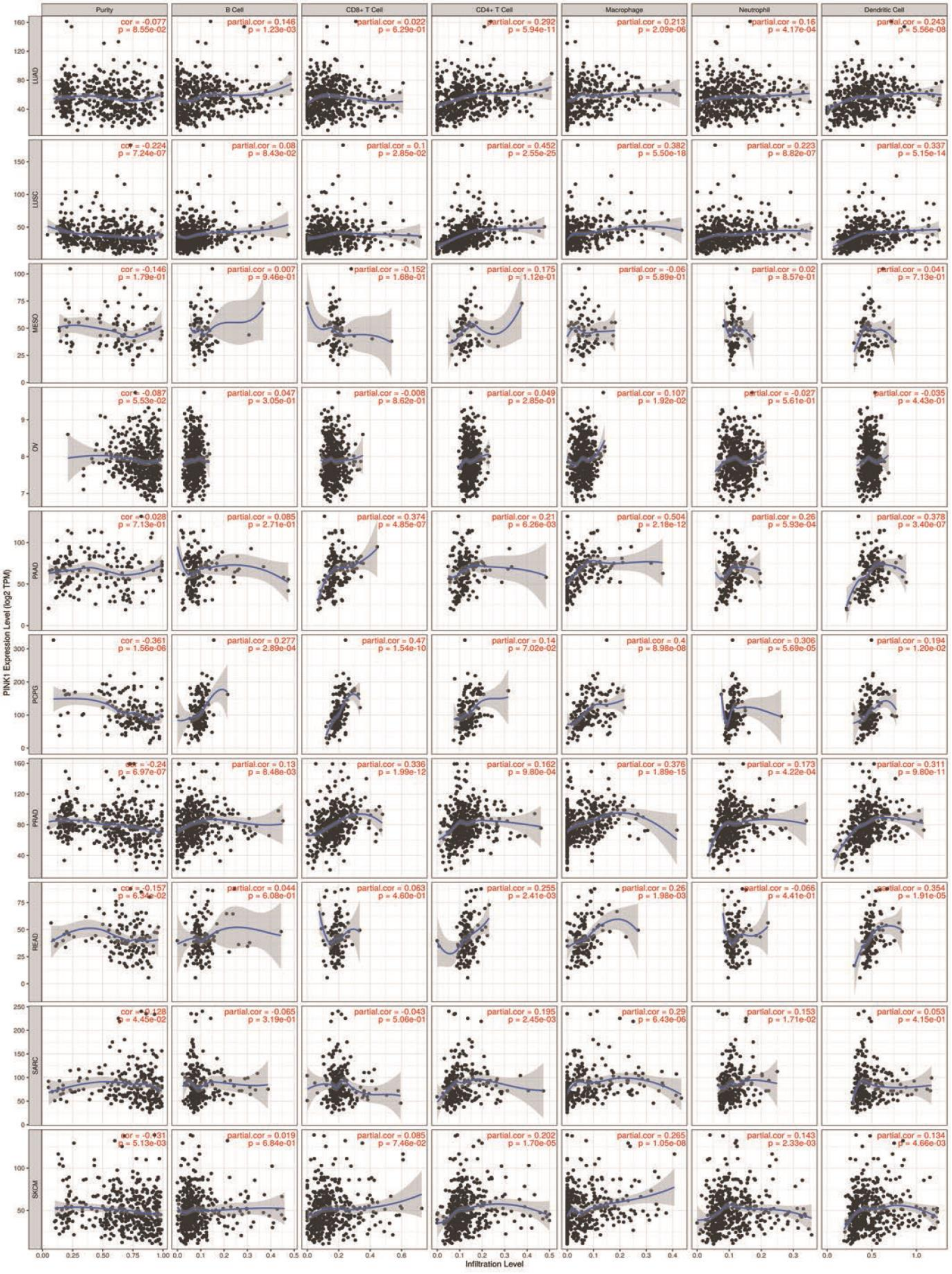
Purity
B Cell
CD8+ T Cell
CD4+ T Cell
Macrophage
Neutrophil
Dendritic Cell
LUAD
LUSC
MESO
OV
PAAD
PCPG
PRAD
READ
SARC
SKCM
PINK1 Expression Level (log2 TPM)
Infiltration Level
cor = -0.077 p = 8.55e-02
partial.cor = 0.146 p = 1.23e-03
partial.cor = 0.022 p = 6.29e-01
partial.cor = 0.292 p = 5.94e-11
partial.cor = 0.213 p = 2.09e-06
partial.cor = 0.16 p = 4.17e-04
partial.cor = 0.243 p = 5.56e-08
cor = -0.224 p = 7.24e-07
partial.cor = 0.08 p = 8.43e-02
partial.cor = 0.1 p = 2.85e-02
partial.cor = 0.452 p = 2.55e-25
partial.cor = 0.382 p = 5.50e-18
partial.cor = 0.223 p = 8.82e-07
partial.cor = 0.337 p = 5.15e-14
cor = -0.146 p = 1.79e-01
partial.cor = 0.007 p = 9.46e-01
partial.cor = -0.152 p = 1.68e-01
partial.cor = 0.175 p = 1.12e-01
partial.cor = -0.06 p = 5.89e-01
partial.cor = 0.02 p = 8.57e-01
partial.cor = 0.041 p = 7.13e-01
cor = -0.087 p = 5.53e-02
partial.cor = 0.047 p = 3.05e-01
partial.cor = -0.008 p = 8.62e-01
partial.cor = 0.049 p = 2.85e-01
partial.cor = 0.107 p = 1.92e-02
partial.cor = -0.027 p = 5.61e-01
partial.cor = -0.035 p = 4.43e-01
cor = -0.028 p = 7.13e-01
partial.cor = 0.085 p = 2.71e-01
partial.cor = 0.374 p = 4.85e-07
partial.cor = 0.21 p = 6.26e-03
partial.cor = 0.504 p = 2.18e-12
partial.cor = 0.26 p = 5.93e-04
partial.cor = 0.378 p = 3.40e-07
cor = -0.361 p = 1.56e-06
partial.cor = 0.277 p = 2.89e-04
partial.cor = 0.47 p = 1.54e-10
partial.cor = 0.14 p = 7.02e-02
partial.cor = 0.4 p = 8.98e-08
partial.cor = 0.306 p = 5.69e-05
partial.cor = 0.194 p = 1.20e-02
cor = -0.24 p = 6.97e-07
partial.cor = 0.13 p = 8.48e-03
partial.cor = 0.336 p = 1.99e-12
partial.cor = 0.162 p = 9.80e-04
partial.cor = 0.376 p = 1.89e-15
partial.cor = 0.173 p = 4.22e-04
partial.cor = 0.311 p = 9.80e-11
cor = -0.157 p = 6.34e-02
partial.cor = 0.044 p = 6.08e-01
partial.cor = 0.063 p = 4.60e-01
partial.cor = 0.255 p = 2.41e-03
partial.cor = 0.26 p = 1.98e-03
partial.cor = -0.066 p = 4.41e-01
partial.cor = 0.354 p = 1.91e-05
cor = 0.128 p = 4.45e-02
partial.cor = -0.065 p = 3.19e-01
partial.cor = -0.043 p = 5.06e-01
partial.cor = 0.195 p = 2.45e-03
partial.cor = 0.29 p = 6.43e-06
partial.cor = 0.153 p = 1.71e-02
partial.cor = 0.053 p = 4.15e-01
cor = -0.131 p = 5.13e-03
partial.cor = 0.019 p = 6.84e-01
partial.cor = 0.085 p = 7.46e-02
partial.cor = 0.202 p = 1.70e-05
partial.cor = 0.265 p = 1.05e-08
partial.cor = 0.143 p = 2.33e-03
partial.cor = 0.134 p = 4.66e-03

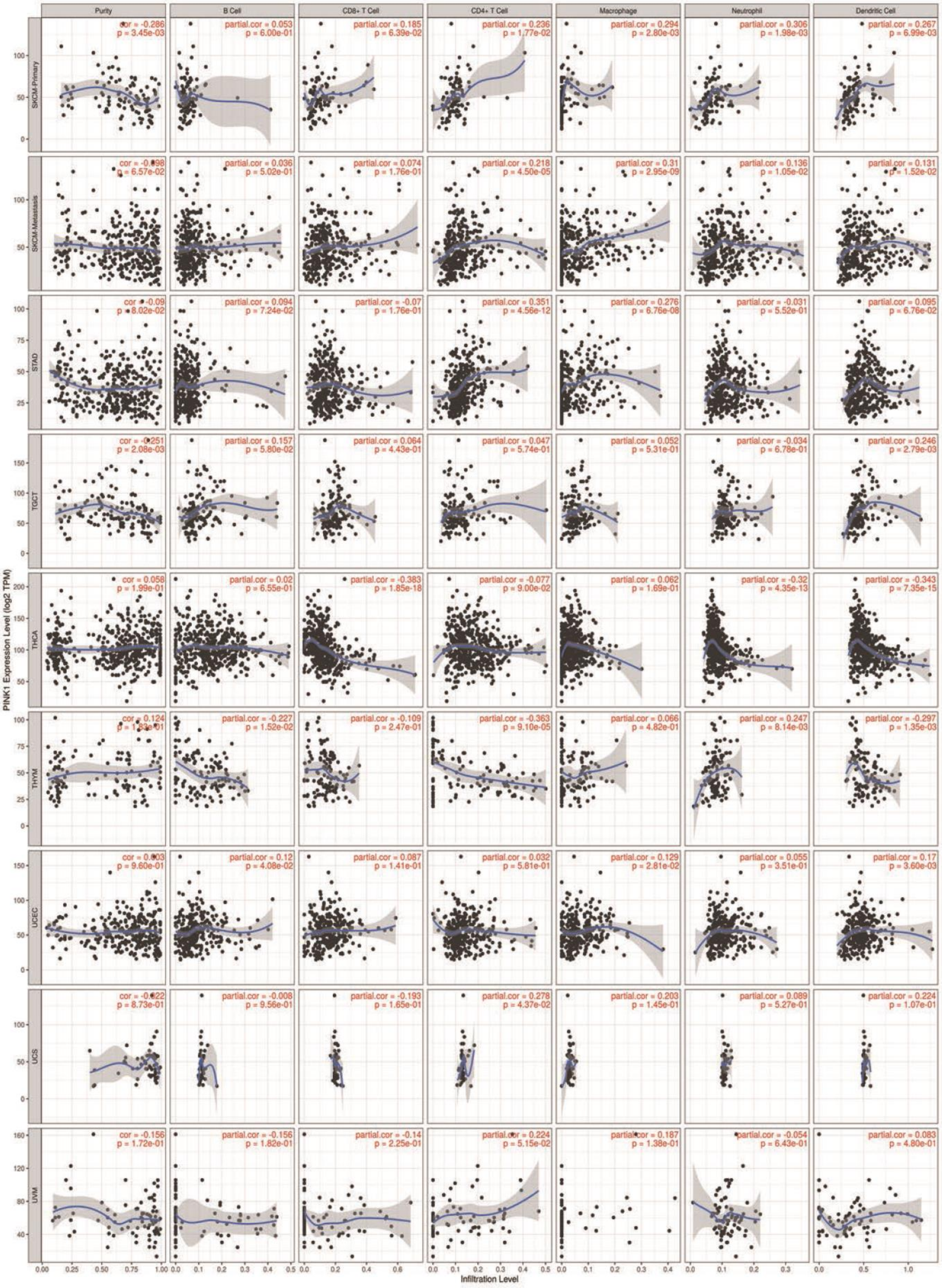

Supplementary Figure 3. The correlation between PINK1 expression and immune infiltration level in pan-cancer based on TIMER database. Abbreviations: ACC, adrenocortical carcinoma; BLCA, bladder urothelial carcinoma; BRCA, breast invasive carcinoma; BRCA-Basal, breast invasive carcinoma-basal; BRCA-Luminal, breast invasive carcinoma-luminal; BRCA-Her2, breast invasive carcinoma-her2; CESC, cervical and endocervical cancer; CHOL, cholangiocarcinoma; COAD, colon adenocarcinoma; DLBC, diffuse large B-cell lymphoma; ESCA, esophageal carcinoma; GBM, glioblastoma multiforme; HNSC, head and neck cancer; HNSC-HPVpos, head and neck cancer-HPV positive; HNSC-HPVneg, head and neck cancer-HPV negative; KICH, kidney chromophobe; KIRC, kidney renal clear cell carcinoma; KIRP, kidney renal papillary cell carcinoma; LGG, lower grade glioma; LIHC, liver hepatocellular carcinoma; LUAD, lung adenocarcinoma; LUSC, lung squamous cell carcinoma; MESO, mesothelioma; OV, ovarian serous cystadenocarcinoma; PAAD, pancreatic adenocarcinoma; PCPG, pheochromocytoma and paraganglioma; PRAD, prostate adenocarcinoma; READ, rectum adenocarcinoma; SARC, sarcoma; SKCM, skin cutaneous melanoma; SKCM-Primary, skin cutaneous melanoma-primary; SKCM-Metastasis, skin cutaneous melanoma-metastasis; STAD, stomach adenocarcinoma; TGCT, testicular germ cell tumors; THCA, thyroid carcinoma; THYM, thymoma; UCEC, uterine corpus endometrial carcinoma; UCS, uterine carcinosarcoma; UVM, uveal melanoma.

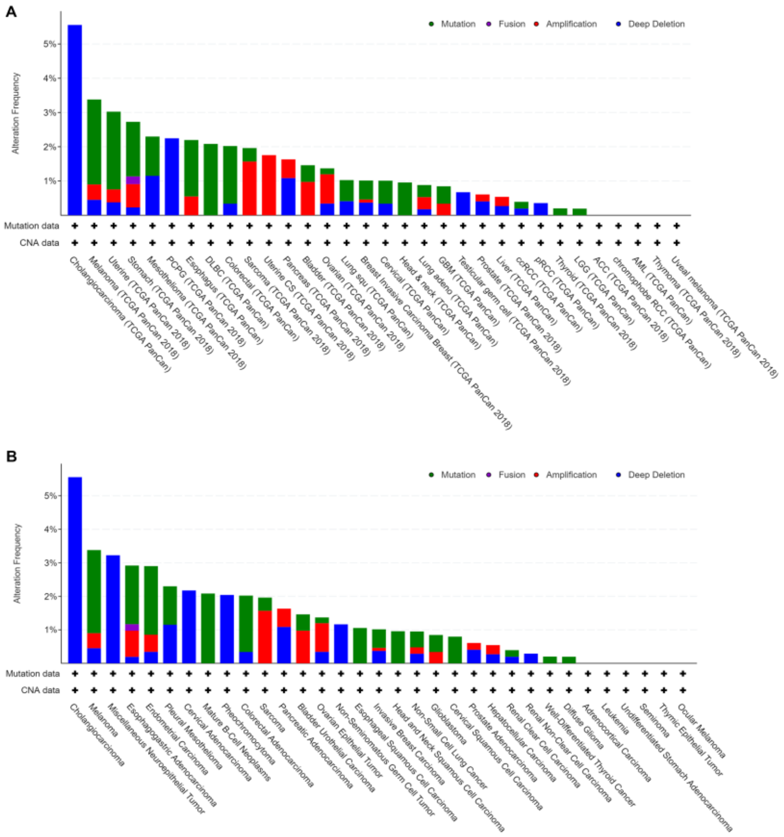

Supplementary Figure 4. The frequency of PINK1 alterations in pan-cancer. (A) according to cancer studies. (B) according to cancer types.

1.2 Supplementary Tables

Supplementary Table 1. Survival analyses of PINK1 expression in pan-cancer (in Kaplan-Meier plotter)

Cancer type	Survival outcome	HR	95%CI	P-value
Bladder carcinoma	OS	0.85	0.63-1.16	0.31
	RFS	0.73	0.36-1.48	0.38
Breast cancer	OS	0.75	0.54-1.05	0.094
	RFS	0.68	0.44-1.06	0.085
Cervical squamous cell carcinoma	OS	1.61	0.97-2.66	0.064
	RFS	1.81	0.82-4	0.14
Esophageal adenocarcinoma	OS	0.38	0.15-0.99	0.039
	RFS	4.27	0.59-31.05	0.12
Esophageal squamous cell carcinoma	OS	0.46	0.2-1.07	0.063
	RFS	1.33	0.51-3.45	0.56
Head-neck squamous cell carcinoma	OS	0.66	0.49-0.90	0.0072
	RFS	0.63	0.3-1.33	0.22
Kidney renal clear cell carcinoma	OS	0.75	0.56-1.01	0.061
	RFS	4.93	0.65-37.65	0.088
Kidney renal papillary cell carcinoma	OS	1.5	0.81-2.78	0.19
	RFS	3.49	1.05-11.61	0.03
Liver hepatocellular carcinoma	OS	0.5	0.35-0.71	8.7e-05
	RFS	0.62	0.44-0.86	0.0039
Lung adenocarcinoma	OS	0.84	0.59-1.18	0.32
	RFS	1.26	0.77-2.04	0.35
Lung squamous cell carcinoma	OS	1.31	0.99-1.72	0.057
	RFS	1.41	0.84-2.36	0.19
Ovarian cancer	OS	0.75	0.55-1.03	0.072
	RFS	0.61	0.4-0.94	0.022
Pancreatic ductal adenocarcinoma	OS	0.58	0.35-0.95	0.03
	RFS	2.56	0.95-6.88	0.056
Rectum adenocarcinoma	OS	1.31	0.57-2.98	0.52
	RFS	3.61	0.41-31.96	0.22
Sarcoma	OS	1.24	0.83-1.85	0.3
	RFS	1.58	0.85-2.96	0.15
Stomach adenocarcinoma	OS	1.31	0.94-1.83	0.11
	RFS	1.52	0.67-3.46	0.32
Testicular germ cell tumor	OS	0.27	0.03-2.72	0.24
	RFS	0.34	0.15-0.82	0.011
Thyroid carcinoma	OS	2.7	1-7.28	0.041
	RFS	0.72	0.31-1.66	0.44
Uterine corpus endometrial carcinoma	OS	2.34	1.54-3.57	4.7e-05
	RFS	1.74	1-3.02	0.046

Abbreviations: HR, hazard ratio; CI, confidence interval; OS, overall survival; RFS, relapse free survival.

Supplementary Table 2. Survival analyses of PINK1 expression in pan-cancer (in GEPIA).

Cancer type	Survival outcome	N	HR	P-value
Total cancer	OS	9500	0.69	0
	DFS	9500	0.82	1.4e-07
Adrenocortical carcinoma (ACC)	OS	76	0.36	0.011
	DFS	76	0.7	0.3
Bladder Urothelial Carcinoma (BLCA)	OS	402	1	0.85
	DFS	402	1.1	0.63
Breast invasive carcinoma (BRCA)	OS	1070	1.2	0.2
	DFS	1070	0.97	0.89
Cervical squamous cell carcinoma and endocervical adenocarcinoma (CESC)	OS	292	0.83	0.43
	DFS	292	0.82	0.49
Cholangio carcinoma (CHOL)	OS	36	2.5	0.052
	DFS	36	1.7	0.23
Colon adenocarcinoma (COAD)	OS	270	0.84	0.47
	DFS	270	0.84	0.49
Lymphoid Neoplasm Diffuse Large B-cell Lymphoma (DLBC)	OS	46	0.38	0.18
	DFS	46	0.17	0.013
Esophageal carcinoma (ESCA)	OS	182	0.97	0.88
	DFS	182	1.6	0.058
Glioblastoma multiforme (GBM)	OS	162	1.2	0.3
	DFS	162	0.87	0.54
Head and Neck squamous cell carcinoma (HNSC)	OS	518	1.3	0.037
	DFS	518	1.4	0.033
Kidney Chromophobe (KICH)	OS	64	1.5	0.53
	DFS	64	0.76	0.67
Kidney renal clear cell carcinoma (KIRC)	OS	516	0.46	7.5e-07
	DFS	516	0.67	0.03
Kidney renal papillary cell carcinoma (KIRP)	OS	282	0.46	0.015
	DFS	282	0.52	0.029
Acute Myeloid Leukemia (LAML)	OS	106	1.1	0.69
	DFS	106	1	1
Brain Lower Grade Glioma (LGG)	OS	514	1	0.99
	DFS	514	1.2	0.16
Liver hepatocellular carcinoma (LIHC)	OS	364	0.94	0.74
	DFS	364	0.75	0.057
Lung adenocarcinoma (LUAD)	OS	478	1	0.96
	DFS	478	0.79	0.13
Lung squamous cell carcinoma (LUSC)	OS	482	1.4	0.0099
	DFS	482	1.7	0.004
Mesothelioma (MESO)	OS	82	0.61	0.043
	DFS	82	0.74	0.3
Ovarian serous cystadenocarcinoma (OV)	OS	424	1.2	0.26
	DFS	424	1	0.79
Pancreatic adenocarcinoma (PAAD)	OS	178	0.84	0.4
	DFS	178	0.85	0.47
Pheochromocytoma and Paraganglioma (PCPG)	OS	182	0.22	0.13
	DFS	182	1.2	0.7
Prostate adenocarcinoma (PRAD)	OS	492	0.94	0.92
	DFS	492	1	0.99
Rectum adenocarcinoma (READ)	OS	92	2.1	0.13
	DFS	92	1.8	0.22
Sarcoma (SARC)	OS	262	1.1	0.77
	DFS	262	0.87	0.44
Skin Cutaneous Melanoma (SKCM)	OS	458	1.1	0.32

	DFS	458	0.98	0.88
Stomach adenocarcinoma (STAD)	OS	384	1	0.77
	DFS	384	1.3	0.24
Testicular Germ Cell Tumors (TGCT)	OS	136	0.36	0.35
	DFS	136	1.2	0.61
Thyroid carcinoma (THCA)	OS	509	1.1	0.91
	DFS	509	0.66	0.16
Thymoma (THYM)	OS	118	0.63	0.53
	DFS	118	0.57	0.22
Uterine Corpus Endometrial Carcinoma (UCEC)	OS	172	0.61	0.17
	DFS	172	1.1	0.74
Uterine Carcinosarcoma (UCS)	OS	56	0.94	0.84
	DFS	56	0.56	0.11
Uveal Melanoma (UVM)	OS	78	1.2	0.66
	DFS	78	1.2	0.71

Abbreviations: HR, hazard ratio; CI, confidence interval; OS, overall survival; DFS, disease free survival.
